# Ethnobotanical Study of Medicinal Plants Used as Anti-Obesity Remedies in the Nomad and Hunter Communities of Burkina Faso

**DOI:** 10.3390/medicines3020009

**Published:** 2016-04-26

**Authors:** Dramane Pare, Adama Hilou, Noufou Ouedraogo, Samson Guenne

**Affiliations:** 1 Laboratory of Biochemistry and Applied Chemistry (LABIOCA), UFR/SVT, 09 BP 848 Ouagadougou 09, University of Ouagadougou, Ouagadougou, Burkina Faso; hiloudio@gmail.com (A.H.); guesams@gmail.com (S.G.); 2 Research Institute for Health Sciences (IRSS) 03 BP 7192 Ouagadougou 03, Ouagadougou, Burkina Faso; ouednouf@gmail.com

**Keywords:** obesity, anorexigenic plant, Burkina Faso, metabolic disease, ethnobotany

## Abstract

**Background**: Obesity is a global epidemic that affects both developed and developing countries. According to World Health Organization (WHO), in 2014, over 1.9 billion adults were overweight. Burkina Faso, like other countries, faces the problem of obesity, with a prevalence of 7.3%. The main cause is excessive intake of caloric foods combined with low physical activity, although genetic, endocrine and environmental influences (pollution) can sometimes be predisposing factors. This metabolic imbalance often leads to multiple pathologies (heart failure, Type II diabetes, cancers, *etc.*). Drugs have been developed for the treatment of these diseases; but in addition to having many side effects, locally these products are not economically accessible to the majority of the population. Burkina Faso, like the other countries bordering the Sahara, has often been confronted in the past with periods of famine during which populations have generally used anorectic plants to regulate their food needs. This traditional ethnobotanical knowledge has not been previously investigated. An ethnobotanical survey was conducted in Burkina Faso in the provinces of Seno (North) and Nayala (Northwest) to list the plants used by local people as an anorectic and/or fort weight loss. **Methods**: The survey, conducted in the two provinces concerned traditional healers, herbalists, hunters, nomads and resourceful people with knowledge of plants. It was conducted over a period of two months and data were collected following a structured interview with the respondents. The approach was based on dialogue in the language of choice of the respondent and the use of a questionnaire. The data have been structured and then statistically analyzed. **Results:** The fifty-five (55) respondents of the survey were aged between 40 and 80 years. Sixty-one (61) plant species, belonging to thirty-one (31) families were listed as appetite suppressants and/or for their anti-obesity properties. The main families of plants are Mimosaceae, Rubiaceae, Asclepiadaceae and Cesalpiniaceae. Fruits are the most used part of the plant organs. Consumption in the raw state or as a decoction are the two main forms of preparation. **Conclusion**: The great diversity of plants cited by informants demonstrates the existence of rich local knowledge to address obesity in Burkina Faso. Evaluation of the biochemical activity of the extracts of the most cited species could allow the development of a phytomedicine economically accessible to the majority of the population. This could allow for the preservation of biodiversity in this region which is weakened by climate change because some of the species cited are in fragile state or are threatened with extinction.

## 1. Introduction

Obesity is a condition that concerns people of all ages in both developed and developing countries. According to the World Health organization (WHO), in 2014, over 1.9 billion adults were overweight in the world. Burkina Faso has faced a fast growing obesity problem in the last decade, and today more than 7.3% of its population is affected [1]. In addition to being a social handicap, this metabolic imbalance is often associated with diseases such as hypertension, myocardial infarction, stroke, type II diabetes, dyslipidemia and certain cancers [2]. Excessive weight gain is usually caused by increased consumption of high caloric foods and decreased physical activity. Genetic (familial predisposition), biological (endocrine disorders), environmental (pollution) [3] factors may also contribute to this problem.

In Burkina Faso as in most developing countries, urbanization and socio-economic development are accompanied by a change in diet towards more with a high energy density foods (more meat, fat, salt and sugary foods) as well as a reduction in physical activity (mechanized transport) [4] resulting in increased storage of the excess calories as fat in adipose tissue. An aggravating cause of the situation in Africa is the antiquated traditional African conception of affluence, according to which obesity of women is a positive indicator of the material abundance of the family and of a good reproductive health. One can find, in the pharmaceutical market, some synthesis of chemical drugs that are used against obesity: Sibutral, Rimonabant, Isomeride, Xenical, Lorcaserin, Ponderal, Alli, and Qsymia. But the cost of these products generally puts them out of reach of most people; and worse, many of these products have many side effects [5].

That is the reason for the withdrawal from the market of some older drugs such as Sibutral, Rimonabant, Isomeride, Ponderal and Xenical [5,6]. The development of new antiobesity molecules from natural products has become a necessity. This seems realizable because in phytotherapy, several types of plants are used against this disease. The plant bioactive extracts would act through their inhibitory activities for digestive lipases, adipocyte differentiation, or by increasing thermogenesis and anorexia [7]. Burkina Faso, like other Sahelian countries, has often been confronted in the past with periods of famine. During these times of food shortage, people have generally used plants with anorectic effects to regulate their food and drink intake. Burkina Faso is also a savannah country with many nomadic and hunter societies. During their displacements or hunting parties, these people could be facing period of lack of food or water. These populations survived thanks to a strong ethnobotanical knowledge able to help in the management of satiety. However, in Burkina Faso there are few data on these plant species used as anorectics or against obesity. An ethnobotanical survey was conducted in the province of Seno (nomadic area in Burkina Faso northern area) and the Nayala (traditional hunting area in northwest of Burkina Faso) in order to collect information on plants used by local people as anorectics and/or to manage weight. This study aimed to establish an inventory of appetite suppressant or antiobesity plant species.

## 2. Materials and Methods

### 2.1. Study Area

**Figure 1:**
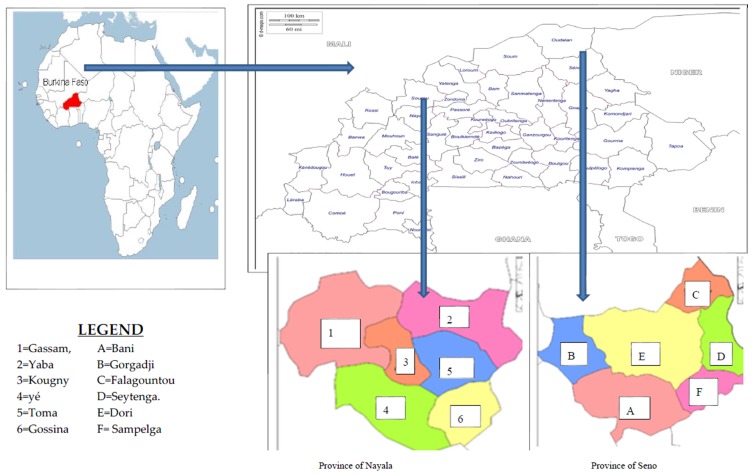
Maps of the survey area.

Burkina Faso (Figure 1) is a landlocked country located in the heart of West Africa and enclosed between six countries: Mali, Niger, Benin, Togo, Ghana and Cote d'Ivoire. It covers an area of approximately 274,000 km². It is located inside the loop of the Niger River between 10 ° and 15 ° north latitude and between 2° east and 5°30’ west longitude. Its capital city is Ouagadougou. The climate is characterized by a long dry season (from October to May) and an irregular rainy season (from June to September). Monthly average temperatures range between 22 °C and 42 °C. Except for the extreme north which consists of desert or semi-desert, Burkina Faso is a savannah country. The homogenous and seasonal-dependent vegetal landscape is constituted by *Parkia biglobosa* (Néré in French), *Vitellaria paradoxa* (Karité in French), *Cassia* sp and *Andasonia digitata* ecosystems. This country is divided into 45 provinces grouped into 13 regions.

The surveys were conducted in the two north provinces where nomadic or hunting populations reside.

Seno Province, whose capital is Dori, is located in the north eastern area of Burkina Faso. It has 215 villages and an area of 6979 km^2^ with a population of 264,815 people [8]. This locality has a Sahelian climate, characterized by a long dry season (May to October) and a short rainy season (average rainfall of 400 mm), with varying temperatures (10–43 °C), low humidity, wind and a large amounts of sunshine, typical of the Sahel. The vegetation is characterized by wooded and shrubby steppe that is heavily damaged. However, there are a few gallery forests which are generally located along the rivers (like the swamp of Dori or the Yakouta River). The dominant types of vegetation are thorn trees [9].

Famine is recurrent in this province. The predominant population is the Fulani group, who are nomadic herders. They have survived drought in this region through their knowledge of appetite suppressing plants.

Located in the northwest of Burkina Faso, Nayala province (whose capital is Toma) has an area of 3919 km^2^ with a population of 156,861 and a northern Sudanian climate. The vegetation consists of shrub or herbaceous savannah with some groves near villages. Soils are clayey [10]. Many hunter groups live in this province. It often happens that these hunters lose themselves in the bush tracking a hunted animal. To survive these situations (temporary lack of water or food, which can take days), they have developed a rich ethnobotanical knowledge on plants possessing appetite suppressing or thirst quenching properties.

### 2.2. Methods

#### 2.2.1. Data Collection

The ethnobotanical survey was conducted in the provinces of Seno and Nayala during the period from August to September 2013. Over 70 interviews were conducted in different localities of these provinces. Data were collected following a structured interview with traditional healers, herbalists and hunters. These groups are located in each of these areas, organized in associations. A preliminary meeting was held during which they were informed about the objectives of the study. After information was provided, the people who agreed to participate in the survey were individually interviewed. The approach was based on a dialogue using the language of choice of the respondent and the use of a questionnaire. A field trip was organized and plants mentioned in the interview were collected with the help of the respondent in order to make the herbal constitution. The intervention of interpreters was necessary in some cases. The cited and harvested plant specimens were identified by Professor Jeanne Millogo-Rasolodimby (Botanist, Department of Ecology/University of Ouagadougou).

#### 2.2.2. Data Analysis

Survey data were first extracted manually then entered and processed by Excel software. The citation frequencies of all data obtained from this study was subjected to descriptive statistical analysis by calculating of frequency of plant citations, using the formula:

(1) $$F=\frac{Number\;of\;people\;who\;cited\;the\;species\;}{Total\;number\;of\;persons\;interviewed}\;\times \;100$$

#### 2.2.3. The Use Value Index (UVI)

The use value index (UVI) of a species for each use class is evaluated to show the importance that people attach to a given species in the localities [11]. It is obtained by calculating the following:

UVI = *U*/*N* (2)

Where *U* is the number of times that species is cited for a category of use and *N* the total number of informants.

## 3. Results

### 3.1. Results

#### 3.1.1. Traditional Knowledge: Age and Gender

**Figure 2:**
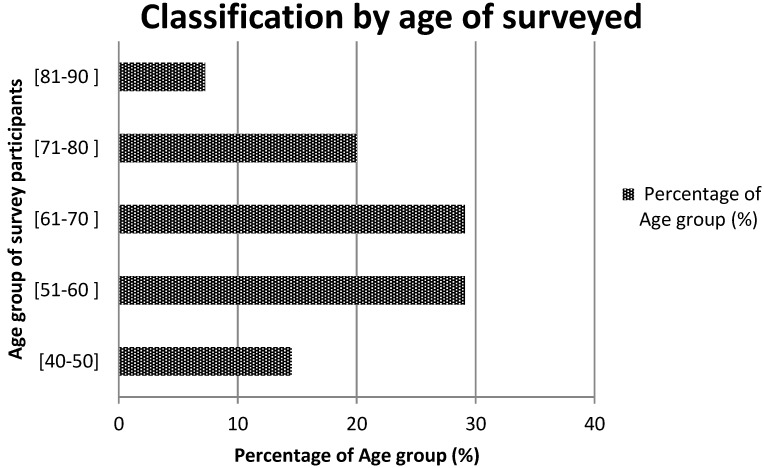
Age group of informants.

During the survey 55 people have been interviewed including 34 in Nayala and 21 in Seno. The mean age varies between 40 and 81 years and over 50% were between 50 and 70 years old (Figure 2). The practice time (in years) varies between 7 and 35. Men represented 92.7% of the respondents *versus* 7.3% what were women. All were traditional healers, herbalists, hunters or elderly nomadic person with knowledge on plants. In total 62 plant species belonging to 32 families were listed as having anorectic and/or anti-obesity activity. Table 1 shows the list and ethno-botanic characteristics of these plant species.

#### 3.1.2. Part of Plant and Method of Preparation

**Figure 3:**
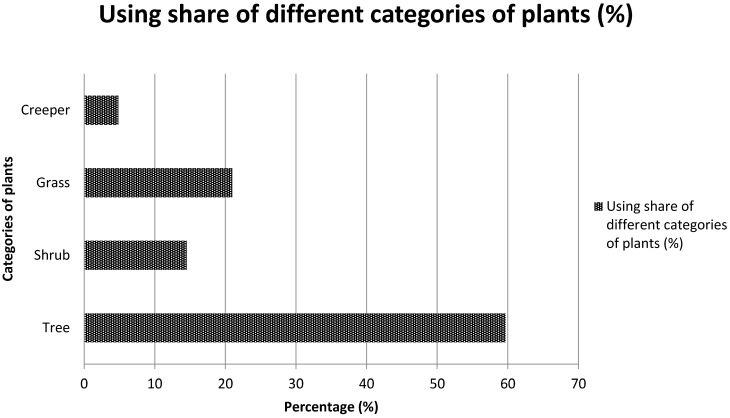
Applied share of different categories of plants.

**Figure 4:**
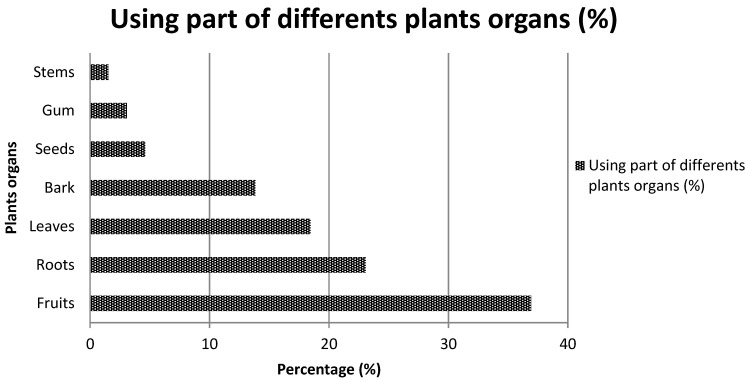
Using part of different plant organs.

**Figure 5:**
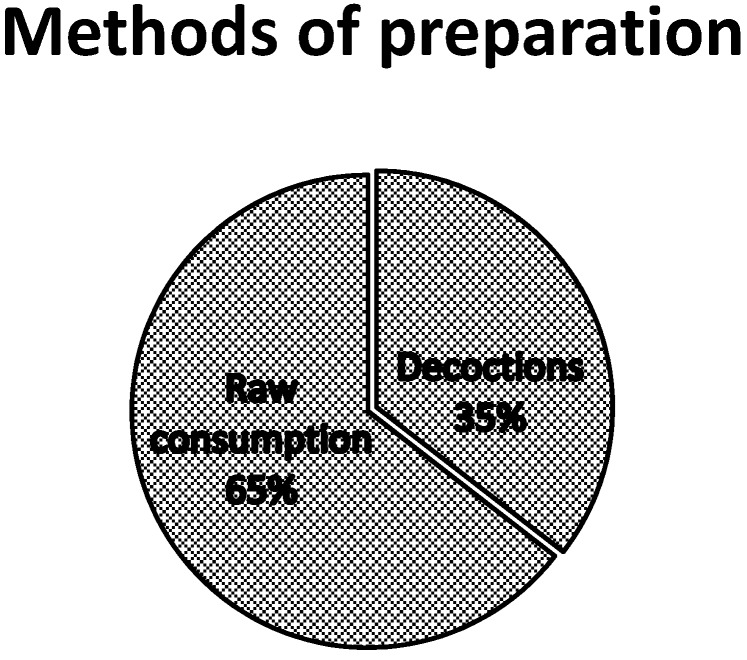
Use of various preparation methods.

The analysis of the mode of use of the various listed plants revealed that 53.6% of plants are appetite suppressants, 30.4% are used to lose weight and 15.9% are used as a thirst quencher. Most of the species cited are trees (59.6%), followed by shrubs (20.9%), herbs (14.5%) and creeping plants (4.8%) (Figure 3). Table 2, Table 3 and Table 4 present the species which are used as appetite suppressants, for weight reduction and as thirst quenchers; with the part used and the use value indexes. *Raphionacme daronii* Berhaut. (Asclepiadaceae) is the most commonly used species for appetite suppressant activity, with a use value index of 0.27; followed by *Gardenia erubescens* Stapf and Hutch. (Rubiaceae) with 0. 22 as its use value index. For slimming property, the species *Tamarindus indica* L. (Caesalpiniaceae) and *Ozoroa insignis* Del. (Anacardiaceae) are the most used with use value of 0.05. *Raphhionacme daronii* Berhaut. (Asclepiadaceae) and *Brachystelma bingeri* A. Chev. (Asclepiadaceae) with respective use value indexes of 0.27 and 0.16 are most commonly used as a thirst quencher. Concerning part of plants, fruits have the highest frequency of use (Figure 4). Decoctions (35.5%) and raw consumption (64.5%) (Figure 5) are the main forms of use. Oral ingestion is the main means of administration.

#### 3.1.3. Families of Plants Used

**Figure 6:**
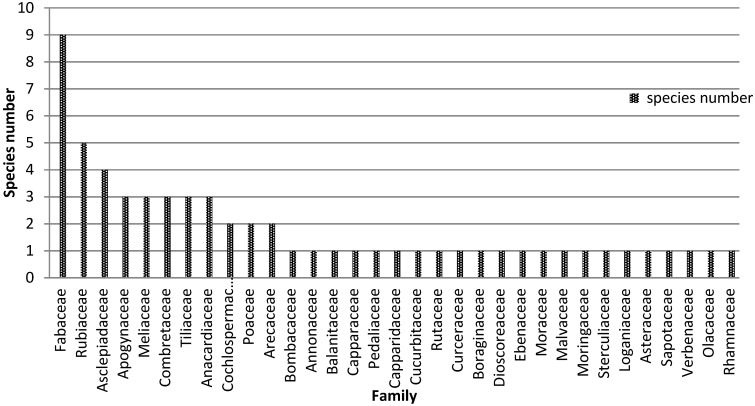
Cited plant families.

The study indicates that Mimosaceae, Rubiaceae, Asclepiadaceae, Cesalpiniaceae, Anacardiaceae, Apogynaceae, Meliaceae, Combretaceae and Tiliaceae have been the most cited as appetite suppressant/anti-obesity plants (Figure 6). *Raphionacme daronii* (*F* = 25.4%), *Gardenia erubescens* (*F* = 20.3%), *Brachystelma bingeri* (*F* = 15.3%), *Commiphora africana* (*F* = 11.9%), *Leptadenia hastata* (*F* = 10. 2%), *Balanites aegyptiaca* (*F* = 10.2%) are the six species with the highest frequencies of use. 

### 3.2. Discussion

The survey has allowed identifying 62 species of plants which have anorectic and/or anti-obesity activity. Most interviewees were men; female healers or hunters are rare in these provinces. In Africa these activities are mainly the responsibility of men and the knowledge is transmitted very often from father to son. The 62 species listed have already been studied for some properties (Table 5).

Eight species, namely *Leptadenia hastata*, *Balanites aegyptiaca*, *Zizyphus mauritiana*, *Tamarindus indica*, *Khaya senegalensis*, *Brachystelma bingeri*, *Azadirachta indica*, and *Adansonia digitata* have been cited both in Nayala and Seno. So, these plants grow well in a Sahelian or in a Sudanian climate. In this study *Raphionacme daronii* (*F* = 25.4%), *Gardenia erubescens* (*F* = 20.3%), *Brachystelma bingeri* (*F* = 15.3%), *Commiphora africana* (*F* = 11.9%) *Leptadenia hastata* (*F* = 10.2%) and *Balanites aegyptiaca* (*F* = 10.16%) are the six species which have presented the highest frequency of citation and greater use value indexes in the group of appetite suppressant plants species. This indicates the importance given to these plants by these populations in the treatment of obesity or as an anorectic.

Raw fresh material directly and decoctions are the two main forms of consumption. Anorectic or thirst quenching plants are usually eaten raw as they are most often used to immediately remedy a situation of hunger or thirst. The preparations generally involve a single plant material, but sometimes mixtures can also be used. In the latter, a synergistic effect may be supposed [12].

There are some differences in the methods of preparation and parts of plants used according to each locality. For example, the decoction of bark and fruits of *Tamarindus indica* is used in Seno while the decoction of roots is used in Nayala. Also, fruits of *Zizuphus mauritiana* are prescribed as an appetite suppressant in both localities but the roots are used for weight loss in Seno.

According to the literature, *Zizyphus mauritiana*, *Tamarindus indica* and *Moringa oleifera* have previously been tested for anti-obesity activity [140,141,142]. This could be linked to a widespread use of these species in many regions for the same indication.

The most cited plants have already been studied for various activities:

*Balan ites aegyptiaca* is mainly consumed in dearth times by the population [143], and it contains carbohydrates, steroidal saponines, fiber, gum [37] alkaloids and flavonoids [32]. It also contains galactose, mannose, arabinose, xylose, rhamnose and glucuronic acid [31]. Their fruits are used against diabetes [30,144] as well as the seeds [145]. The plant is also known having anti-tumor activity [33] and an anti-infertility property [146].

Leaves of *Leptadenia hastata* are rich in tannins, glycosides, alkaloids, carbohydrates and flavonoids. [98]. d-Cymarose and d-oleandrose were also isolated [97]. They are used against diabetes 97] and supposed havingantibacterial activity [98] and anti-androgen property [99].

*Commiphora africana* contains cardiac glycosides and reducing sugars [147]. It has antimicrobial activity and is traditionally used against diarrhea [54]. 

Fruit and young leaves of *Gardenia erubescens* are consumed during dearth periods [148]. These fruits contain carbohydrates and fibers [149] and they are also rich in anthraquinones, tannins, sterols and triterpenes [77]. The leaves contain tannins, triterpene saponins, other triterpenoids, iridoids and sterols. The bark is rich in triterpene saponins, triterpenoids and sterols [37]. The leaves are used for the treatment of digestive parasites in small ruminants [78] and the bark of the trunk has analgesic and diuretic activity [79].

*Gardenia erubescens* is traditionally used against hepatitis [150].

*Raphionacme daronii* is a plant used during times of famine; it is eaten raw [148,151]. The tuber contains sugars and starch [114].

The tuber of *Brachystelma bingeri* is used against insufficient sperm and male sexual asthenia, and it is very nutritious, stimulating and can act as a tonic [37]. It is consumed during famine periods [143] and is rich in carbohydrates, saponins, triterpenes and sterols [37].

The most cited plants listed during the survey have not been investigated for an anti-obesity study. So, there is a need to test their bioactivity and eventually study the phytochemistry and pharmacological profile of these plants in order to scientifically support traditional ethnobotanical and to secure their use. 

## 4. Conclusions

The ethnobotanical survey revealed the presence of an enormous biodiversity of plants used in these two north provinces of Burkina Faso to modulate appetite and thirst. This rich ethnobotanical background indicates the high potential of traditional knowledge to serve for the development of natural product-derivates as affordable medicines. This may contribute to the preservation of traditional knowledge on anti-obesity herbs of these two provinces of Burkina Faso. Twenty-two species cited are in fragile state or are threatened with extinction. This requires taking safeguard measures. It is therefore useful to study the ecology of these species, evaluate the resources and the natural regeneration potential. Reforestation with these species requires the mastery of the production of seeds and planting in areas of high use. This is an important endeavour that could help to fight against the massive destruction of these plants, in the context of climate change and the unprecedented human pressure on the environment. Investigation into these six most cited and not yet studied species could lead to the discovery of new products to address the obesity epidemic.

## Figures and Tables

**Table 1:** Plants listed after the survey in Seno and Nayala.

Species and Family	Local Name	Frequency Citation (%)	Parts Used	Indication	Preparation and Use Methods	Posology
1. ***Acacia laeta* Benth. (Fabaceae)**	Gon sablega (mo)	**1.7**	bark	weight loss	decoction	ND
2. ***Acacia nilotica*(L.) Delile (Fabaceae)**		**1.7**	bark	weight loss	decoction	ND
3. ***Acacia senegal* (L.) Willd. *(Fabaceae)***	Gommier (Fran)	**3.4**	gum	appetite suppressant and thirst quencher	raw gum consumption	ND
4. ***Acacia seyal* Del. (Fabaceae)**	Gon-miougou	**1.7**	bark	weight loss	decoction	ND
5. ***Adansonia digitata* L. (Bombacaceae)**	Baobab (fran)	**3.4**	root and bark	thirst quencher	raw root and bark consumption	ND
6. ***Afzelia africana* Smith ex Pers. (Fabaceae)**	Para (san)	**1.7**	leaves	weight loss	decoction of a mixture of *afzelia africana* leaves and roots of *cochlospermum tinctorium* is used as a drink	ND
7. ***Annona senegalensis* Pers. (Annonaceae)**	Guinikou (san)	**3.4**	fruits	appetite suppressant	raw fruits consumption	ND
8. ***Azadirachta indica* A. Juss. (Meliaceae)**	Kakki (ful)	**1.7**	leaves	weight loss	decoction of the bark and use as a drink before meal	ND
9. ***Balanites aegyptiaca* (L.) Delile (Balanitaceae)**	Tanèè (ful), sinbèlè (san)	**10.16**	fruits	appetite suppressant	raw fruits consumption	ND
10. ***Bauhinia rufescens* Lam. (Fabaceae)**	Tipoiga (mo)	**3.4**	bark	weight loss	decoction	ND
11. ***Boscia angustifolia* A. Rich. (Capparaceae)**	Haasu carè (sonrai)	**3.4**	young leaves	weight loss	reduce young dried leaves in powder and mix this powder with porridge and drink	ND
12. ***Brachystelma bingeri* A.Chev. (Asclepiadaceae)**	Sensenega (mo), Daffio (tmac)	**15.25**	roots	appetite suppressant and thirst quencher	raw root consumption	ND
13. ***Cadaba farinosa* Forsk. (Capparidaceae)**	Moussilèè (san)	**3.4**	leaves	appetite suppressant	raw leaves consumption	Causes fart
14. ***Ceratotheca sesamoides* Endl. (Pedaliaceae)**	Dou (san)	**6.78**	leaves	appetite suppressant and thirst quencher	raw leaves consumption	ND
15. ***Ceropegia senegalensis* H. (Apocynaceae)**	Kirimougoin (san)	**3.4**	roots	appetite suppressant	raw root consumption	ND
16. ***Citrullus colocynthis* (L.) Schrad. (Cucurbitaceae)**	Dènè (ful)	**8.47**	fruits	appetite suppressant and thirst quencher	raw fruits consumption	ND
17. ***Citrus aurantiifolia* (Christm.) Swingle (Rutaceae)**		**3.4**	fruits	weight loss	decoction of the leaves of *combretum micranthum* then add fruit juice of *citrus aurantifolia* and use as a drink	1 liter per day for 2 to 3 months
18. ***Cochlospermum planchonii* Hook. f. ex Planch. (Cochlospermaceae)**	Biripin (san)	**3.4**	roots	weight loss	decoction	ND
19. ***Cochlospermum tinctorium* Perrier ex A. Rich. (Cochlospermaceae)**	Gotoro (san)	**1.7**	roots	weight loss	decoction of *afzelia africana* leaves and roots of *cochlospermum tinctorium* and use as a drink	ND
20. ***Combretum micranthum* G. Don. (Combretaceae)**	Randega (mo)	**1.7**	leaves	weight loss	decoction of the leaves of *combretum micranthum* then add the fruit juice of *citrus aurantifolia* and use as a drink	1 liter per day for 2 to 3 months
21. ***Commiphora africana* (A. Rich.) Endl. (Curceraceae)**	Nbadadi (ful)	**11.86**	roots	appetite suppressant and thirst quencher	raw roots consumption	ND
22. ***Cordia africana* Lam. (Boraginaceae)**		**1.7**	fruits	appetite suppressant	raw fruits consumption	ND
23. ***Detarium microcarpum* Guill. et Perr. (Fabaceae)**	Koro (san)	**3.4**	fruits	appetite suppressant	raw fruits consumption	ND
24. ***Digitaria exilis* (Kippist) Stapf (Poaceae)**	Pii (san)	**1.7**	seeds	weight loss	cooking and eat	ND
25. ***Dioscorea bulbifera* L. (Dioscoreaceae)**	Kou (san)	**1.7**	roots	appetite suppressant	raw root consumption	ND
26. ***Diospyros mespiliformis* Hochst. ex A.DC (Ebenaceae)**		**1.7**	fruits	appetite suppressant	raw fruits consumption	ND
27. ***Entada africana* Guill et Perr. (Fabaceae)**	Séremanan (san)	**1.7**	leaves	weight loss	decoction	ND
28. ***Fadogia agrestis* Schweinf. ex Hiern (Rubiaceae)**		**1.7**	roots	appetite suppressant	raw consumption	ND
29. ***Ficus sycomorus* L. (Moraceae)**	Goro (san)	**3.4**	fruits	appetite suppressant	raw fruits consumption	ND
30. ***Gardenia aqualla* Stapf & Hutch (Rubiaceae)**	Kowin (san)	**1.7**	fruits	appetite suppressant	raw fruits consumption	ND
31. ***Gardenia erubescens* Stapf & Hutch (*Rubiaceae)***	Kouin (san)	**20,34**	fruits	appetite suppressant	raw fruits consumption	ND
32. ***Grewia bicolor* Juss. (*Tiliaceae)***	Keli (ful)	**1.7**	fruits	appetite suppressant	raw fruits consumption	ND
33. ***Grewia flavescens* Juss. (Tiliaceae)**		**1.7**	fruits	appetite suppressant	raw fruits consumption	ND
34. ***Grewia villosa* Willd. (Tiliaceae)**	Kiborlohi (ful)	**1.7**	fruits	appetite suppressant	raw fruits consumption	1 glass of fruit has anorectic effects for a half day
35. ***Hibiscus sabdariffa* L. (Malvaceae)**	Fouon (san)	**5.08**	seeds	appetite suppressant	decoction	ND
36. ***Holarrhena floribunda* (G.Don) T.Durand & Schinz var. *florinbunda (*Apocynaceae)**	Ninogga (mo)	**1.7**	leaves	weight loss	decoction	ND
37. ***Hyphaene thebaica* (L.) Mart (Arecaceae)**	Gelohi (ful)	**1.7**	fruits	appetite suppressant	raw fruits consumption	ND
38. ***Khaya senegalensis* (Desv.) A.Juss. (Meliaceae)**	Kuka (mo)	**1.7**	bark	weight loss	decoction	ND
39. ***Lannea microcarpa* Engl. & K. Krause (Anacardiaceae)**	Touo (san)	**1.7**	fruits	appetite suppressant	raw fruits consumption	ND
40. ***Leptadenia hastata* Vatke (Asclepiadaceae)**	Toun (sa) Tatola (tmac)	**10.16**	leaves and fruits	appetite suppressant	raw fruits consumption	ND
41. ***Mitragyna inermis* (Willd.) Kuntze (Rubiaceae)**	Kadioli (ful)	**1.7**	leaves and bark	weight loss	decoction of the bark or leaves and use as a drunk	ND
42. ***Moringa oleifera* Lam (Moringaceae)**	Basankoé (san)	**1.7**	principal root	weight loss	make a decoction of the dried primary root and use as a drink	ND
43. ***Ozoroa insignis* Del. (Anacardiaceae)**	Bouwélèbondan (san)	**5.08**	leaves	weight loss	decoction of the leaves and use for drinking and washing	ND
44. ***Panicum laetum* Kunth (Poaceae)**		**1.7**	seeds	appetite suppressant	decoction	ND
45. ***Parkia biglobosa* (Jacq.) G.Don (Fabaceae)**	Koussi (san)	**3.4**	bark, root	weight loss	decoction of the roots of *ximenia americana* and bark of *parkia biglobosa*, drinking and wash with decoction	ND
46. ***Phoenix dactylifera* L. (Arecaceae)**	Datte (fran)	**1.7**	fruits	appetite suppressant	raw fruit consumption	ND
47. ***Pseudocedrela kotschyi* (Schweinf.) Harms (Meliaceae)**	Siguédré (mo)	**1.7**	small branches	thirst quencher	raw small branches consumption	ND
48. ***Raphionacme daronii* Berhaut (Asclepiadaceae)**	Goin (sa)	**25.42**	root	appetite suppressant and thirst quencher	raw root consumption	ND
49. ***Saba senegalensis* (A.DC.) Pichon var. *senegalensis (*Apocynaceae)**	Mara (san)	**3.4**	fruits	appetite suppressant	raw fruits consumption	ND
50. ***Sarcocephalus latifolius (*Sm.) E.A.Bruce (Rubiaceae)**		**3.4**	fruits	appetite suppressant	raw fruits consumption	ND
51. ***Sclerocarya birrea* (A.Rich.) Hochst. (Anacardiaceae)**	Nobga (Mo)	**3.4**	fruits	thirst quencher	raw fruits consumption	ND
52. ***Sterculia setigera* Delile (Sterculiaceae)**		**1.7**	gum	appetite suppressant	raw gum consumption	ND
53. ***Strychnos spinosa Lam.* Lam (Loganiaceae)**	Kartountoun (sa)	**3.4**	fruits	appetite suppressant and thirst quencher	raw fruits consumption	Excess fruit gives stomach bloating
54. ***Tamarindus indica* L. (Fabaceae)**	Inguètabi (ful)	**5.08**	bark, fruits root	weight loss	1. decoction of the bark powder and dried fruit and use as drink 2. decoction of the roots and use as a drink	Take half glass of tea decoction in the morning and noon before meals
55. ***Terminalia avicennioides* Guill. & Perr. (Combretaceae)**	Sounsoun (san)	**1.7**	root	thirst quencher	raw roots consumption	ND
56. ***Terminalia* macroptera Guill. & Perr. (Combretaceae)**	Kouon (san)	**3.4**	young leaves	appetite suppressant	raw leaves consumption	ND
57. ***Vernonia kotschyana* Sch.Bip. ex Walp. (Astéraceae)**	Yirimassa (diou)	**5.08**	root	appetite suppressant	raw root consumption (fresh or dried)	
58. ***Vitellaria paradoxa*C.F.Gaertn. (Sapotaceae)**	Kouu (san)	**5.08**	fruits	appetite suppressant	raw fruits consumption	ND
59. ***Vitex doniana* Sweet (Verbenaceae)**	Koutiin (san)	**3.4**	fruits	appetite suppressant	raw fruits consumption	ND
60. ***Ximenia americana* L. (Olacaceae)**	Marafoo (san)	**1.7**	root	weight loss	decoction of the roots of *ximenia americana* and bark of *parkia biglobosa*, drink and wash with decoction	ND
61. ***Xysmalobium heudelotianum* Decne. (Asclepiadaceae)**	Kirimougoin (san)	**1.7**	root	appetite suppressant	raw root consumption	ND
62. ***Zizyphus mauritiana* Lam. (Rhamnaceae)**	Guiabè (ful), tomon (san)	**6.78**	fruits, root	appetite suppressant	raw fruits consumption, boil the roots and take the decoction beverage	Take ½ glass of decoction on morning and evening

**Table 2:** Species with supposed appetite suppressant activity, their use value index and the parts used.

Species	Use value Index	Parts Used
1. *Annona senegalensis*	0.036	Fruits
*2. Balanites aegyptiaca*	0.109	Fruits
*3. Brachystelma bingeri*	0.163	Tuber
*4. Cadaba farinosa*	0.036	Leaves
*5. Ceratotheca seésamoiïdes*	0.072	Leaves
*6. Ceropeogia senegalensis*	0.036	Roots
*7. Citrullus colocynthis*	0.090	Fruits
*8. Commiphora africana*	0.127	Bark
*9. Cordia africana*	0.018	Fruits
*10. Detarium microcarpum*	0.036	Fruits
*11. Dioscorea bulbifera*	0.018	Tuber
*12. Diospyros mespiliformis*	0.018	Fruits
*13. Fadogia agrestis*	0.018	Roots
*14. icus sycomorus*	0.036	Fruits
*15. Gardenia aqualla*	0.018	Fruits
*16. Gardenia erubescens*	0.218	Fruits
*17. Grewia bicolor*	0.018	Fruits
*18. Grewia flavescens*	0.018	Fruits
*19. Grewia villosa*	0.018	Fruits
*20. Hibiscus sabdariffa*	0.054	Seeds
*21. Hyphaene thebaica*	0.018	Fruits
*22. Lannea microcarpa*	0.018	Fruits
*23. Leptadenia hastata*	0.109	Leaves
*24. Panicum laetum*	0.018	Seeds
*25. Phoenix dactylifera*	0.018	Fruits
*26. Raphionacme daronii*	0.272	Tuber
*27. Sarcocephalus latifolus*	0.036	Fruits
*28. Sclerocarya birrea*	0.036	Fruits
*29. Sterculia setigera*	0.036	Gum
*30. Strychnos spinosa*	0.018	Fruits
*31. Saba senegalensis*	0.018	Fruits
*32. Terminalia macroptera*	0.036	Leaves
*33. Tamarindus indica*	0.054	Fruits
*34. Vernonia kotschyana*	0.054	Roots
*35. Vitellaria paradoxa*	0.054	Fruits
*36. Vitex doniana*	0.036	Fruits
*37. Xysmalobuium heudelotianum*	0.018	Tuber
*38. Zizyphus mauritiana*	0.072	Fruits

**Table 3:** Plants having weight reduction potential, their use value index and the parts used.

Species	Use Value Index	Parts Used
1. *Acacia laeta*	0.018	Bark
*2. Acacia nilotica*	0.018	Bark
*3. Acacia seyal*	0.018	Bark
*4. Afzelia africana*	0.018	Leaves
*5. Azadirachta indica*	0.018	Leaves
*6. Bauhinia rufescens*	0.036	Bark
*7. Boscia angustifolia*	0.036	Young leaves
*8. Citrus aurantiifolia*	0.036	Fruits
*9. Cochlospermum planchonii*	0.036	Root
*10. Cochlospermum tinctorium*	0.018	Root
*11. Combretum micranthum*	0.018	Leaves
*12. Digitaria exilis*	0.018	Seeds
*13. Entada africana*	0.018	Leaves
*14. Holarrhena floribunda*	0.018	Leaves
*15. Khaya senegalensis*	0.018	Bark
*16. Mitragyna inermis*	0.018	Leaves and bark
*17. Moringa oleracea*	0.018	Root
*18. Ozoroa insignis*	0.054	Leaves
*19. Parkia biglobosa*	0.036	Bark and seeds
*20. Tamarindus indica*	0.054	Bark and fruits
*21. Ximenia americana*	0.018	Root

**Table 4:** Thirst quencher Species with supposed thirst quenching activity, their usual value an the parts used.

Species	Use Value Index	Parts Used
1. *Acacia senegal*	0.036	Gum
*2. Adansonia digitata*	0.036	Bark
*3. Brachystelma bingeri*	0.163	Tuber
*4. Ceratotheca sesamoides*	0.072	Leaves
*5. Citrullus colocynthis*	0.090	Fruits
*6. Commiphora africana*	0.127	Bark
*7. Pseudocedrela kotschyi*	0.018	Stem
*8. Raphionacme daronii*	0.272	Tuber
*9. Sclerocarya birrea*	0.036	Fruits
*10. Strychnos spinosa*	0.036	Fruits
*11. Terminalia avicennioides*	0.018	Seeds

**Table 5:** Pytocemistry and pharmacoloy of plants cited.

Species and family	Wild or Cultivated Status	Availability Information/Threat Status	Phytochemistry	Pharmacological properties
***1. Acacia laeta* Benth. (Fabaceae)**	wild	available species	carbohydrate [13]	anti microbial activity [14]
***2. Acacia nilotica*(L.) Delile (Fabaceae)**	wild	available species	alkaloids, glycosides, anthraquinones, cardiac glycosides [15]	antiplasmodial activity [16], antidiabetic activity [17]
***3. Acacia senegal* (L.) Willd. (Fabaceae)**	wild	available species	alkaloids, glycosides, flavonoids [18]	antidiabetic activity [19] hepatoprotective activity [20]
***4. Acacia seyal* Del. (Fabaceae)**	wild	available species	proteins, phenolics, flavonoids and anthocyanin [13]	antibacterial activities [21]
***5. Adansonia digitata* L. (Bombacaceae)**	wild	threatened species	protein, carbohydrate, fat, fibre, ash, vitamin C, A [22]	antidiarrhoeal activity [23],anti-tumor action [24]
***6. Afzelia africana* Smith ex Pers. (Fabaceae)**	wild	threatened species	lipide, carbohydrate [25] alkaloids [26]	antidiabetic and haematological effect [27] anthelmintic effect [26]
***7. Annona senegalensis* Pers. (Annonaceae)**	wild	available species	rutin, quercetin, quercetrin, anonaïne, tannin glycosides, proteins [27]	anticonvulsant properties [28]
***8. Azadirachta indica* A. Juss. (Meliaceae)**	wild/cultivated	available species	reducing sugar, glycosides, alkaloids, tannins, flavonoids, terpenoids, saponin [29]	antibacterial activity [29]
***9. Balanites aegyptiaca* (L.) Delile (Balanitaceae)**	wild	available species	galactose, mannose, arabinose, xylose, rhamnose [30]; alkaloids, flavonoids [31]; saponoside steroidal [32]	anti-tumor activity [33]
***10. Bauhinia rufescens* Lam. (Fabaceae)**	wild	available species	carbohydrate, crude fibre, crude proteins cyanogenic glucoside [34] menisdaurin, oxepin [35]	antibacterial effects [34]
***11. Boscia angustifolia* A. Rich. (Capparaceae)**	wild	threatened species	alkaloids and saponins [36]	antibacterial activity [36]
***12. Brachystelma bingeri* A.Chev. (Asclepiadaceae)**	wild	threatened species	saponins, triterpens, sterols [37]	treatment of insufficient sperm, male sexual asthenia, as tonicorstimulant [37]
***13. Cadaba farinosa* Forsk. (Capparidaceae)**	wild	available species	cadabicine [38]	
***14. Ceratotheca sesamoides* Endl. (Pedaliaceae)**	wild/cultivated	available species	flavonolignans, triterpene saponins, isoflavones, triterpenoids [37] and phenylpropanoid lignan [39]	antiplasmodial activity [40]
***15. Ceropegia senegalensis* H. (Apocynaceae)**	wild	available species		
***16. Citrullus colocynthis* (L.) Schrad. (Cucurbitaceae)**	wild/cultivated	available species	β-sitosterol [41]	antidiabetic effect [42] analgesic activities [43]
***17. Citrus aurantifolia* (Christm.) Swingle (Rutaceae)**	cultivated	available species	5-geranyloxypsoralen; 5-geranyloxy-7-methoxycoumarin; 5,7-dimethoxycoumarin; 5-methoxypsoralen; and 5,8-dimethoxypsoralen [44]	anti-cancer activity [45], anti-mycobacterium tuberculosis activity [44]
***18. Cochlospermum planchonii* Hook. f. ex Planch. (Cochlospermaceae)**	wild	available species	cardiac glycosides, cardenolides and dienolides, alkaloids, steroids, and tannins, flavonoid, phlobatannins [46]	anti-ulcerogenic activity [47]
***19. Cochlospermum tinctorium* Perrier ex A.Rich. (Cochlospermaceae)**	wild	available species	alkaloids, flavonoids, tannins and cardiac glycoside [48]	antimicrobial activity [49], hepatoprotective activity [50]
***20. Combretum micranthum* G. Don. (Combretaceae)**	wild	threatened species	epicatechin and catechin as penta-acetates; epigallocatechin, gallocatechin and bartogenic acid 28-β-d-glucoside [51]	antihyperglycaemic activity [52], antibacterial [53]
***21. Commiphora africana* (A. Rich.) Endl. (Curceraceae)**	wild	threatened species	cardiac glycosides [54]; α-oxobisabolene [55]	antimicrobial activity [54]
***22. Cordia africana* Lam. (Boraginaceae)**	wild	available species	alkaloids, flavonoids, total phenols and tannins [56]	antibacterial activities [57])
***23. Detarium microcarpum* Guill. et Perr. (Fabaceae)**	wild	threatened species	phenols, flavonoids, saponins, triterpenes/steroids and glycosides [58]	against hepatitis c virus [59]
***24. Digitaria exilis* (Kippist) Stapf (Poaceae)**	wild	available species	apigenin and luteolin [60]	postprandial hyperglycemia [61]
***25. Dioscorea bulbifera* L. (Dioscoreaceae)**	cultivated	available species	carbohydrates, proteins, amino acids, fats, oils, steroids, glycosides, alkaloids, tannins and phenolics [62]	antifungal actions [63] antibacterial activities [64]
***26. Diospyros mespiliformis* Hochst. ex A.DC (Ebenaceae)**	wild	threatened species	flavonoids [65]	antipyretic, analgesic and anti-inflammatory [66]
***27. Entada africana* Guill et Perr. (Fabaceae)**	wild	available species	alkaloids, saponins, flavonoids, glycosides, anthraquinone, terpenes, phenols, resins and saponins [67]	anti-angiogenic activity [68] anti-hepatitis C [69]
***28. Fadogia agrestis* Schweinf. ex Hiern (Rubiaceae)**	wild	available species	monoterpene glycosides [70]	analgesic and anti-inflammatory effects [71] antidiabetic [72]
***29. Ficus sycomorus* L. (Moraceae)**	wild	available species	tannins, alkaloids, reducing compounds, saponins, flavonoids, steroid, terpenoids and anthracenoside [73]	sedative and anticonvulsant effects [74]
***30. Gardenia aqualla* Stapf & Hutch (Rubiaceae)**	wild	available species	flavonoids, phytosterols, phenolics, carbohydrates, tannins, triterpenoids, antraquinone [75]	anticancer activities [76] antimicrobial activity [75]
***31. Gardenia erubescens* Stapf & Hutch (Rubiaceae)**	wild	threatened species	fibers [77] anthraquinons, tannins, sterols and triterpens. [78]	analgesic and diuretic activity. [79].
***32. Grewia bicolor* Juss. (Tiliaceae)**	wild	available species	β-sitosterol, triterpene ester, triterpenes lupeol and betulin, beta-sitosterol glucoside, harmane, 6-methoxyharmane and 6-hydroxyharmane [80]	anticonvulsant and anxiolytic properties [81]
***33. Grewia flavescens* Juss. (Tiliaceae)**	wild	available species	flavonoids, phytosterols, phenolics, carbohydrates, tannins, triterpenoids [82]	anti-diabetic [82]
***34. Grewia villosa* Willd. (Tiliaceae)**	wild		triterpenoids, steroids, glycosides, flavones, lignanes, phenolics, alkaloids, lactones [83]	anti-bacterial and analgesic effect [83]
***35. Hibiscus sabdariffa* L. (Malvaceae)**	cultivated	available species	alkaloids, tannins, saponnins, glycosides, phenols and flavonoids, glycosides [84]	diuretic activity [85] anti-obesity effects [86]
***36. Holarrhena floribunda (*G.Don) T.Durand & Schinz var. *florinbunda (*Apocynaceae)**	wild	available species	fat, fiber, protein, carbohydrates, alkaloid, saponin, tannin and cardiac glycosides [87]	hypoglycaemic activity [88]
***37. Hyphaene thebaica* (L.) Mart (Arecaceae)**		available species	tannins, steroids and moderate level of saponins, carbohydrates, cardiac glycosides, flavonoids, and terpinoids [89]	antimicrobial properties [90] hypoglycaemic properties [89]
***38. Khaya senegalensis* (Desv.) A.Juss. (Meliaceae)**	wild	threatened species	alkaloids, saponins, tannins and flavonoids [91]	hepatoprotective activity [92]
***39. Lannea microcarpa* Engl. & K. Krause (Anacardiaceae)**	wild	threatened species	anthocyanosides [93] 4α-methoxy-myricetin 3-α-l-rhamnopyranoside, myricetin 3-α-l-rhamnopyranoside, myricetin 3-β-d-glucopyranoside, vitexin, isovitexin, gallic acid and epi-catechin [94]	anti-inflammatory activities [95]
***40. Leptadenia hastata* Vatke (Asclepiadaceae)**	wild	available species	d-cymarose and d-oleandrose [96]. tannins, glycosides, alkaloids, flavonoids. [97]	diabetes [96] antibacterial activity [98] anti-androgen property [99].
***41. Mitragyna inermis* (Willd.) Kuntze (Rubiaceae)**	wild	available species	sterol, triterpene, polyphenol, flavonoïd, catechic tannin, saponoside and alkaloid [100]	anticonvulsant properties [101] hepatoprotective activity [102]
***42. Moringa oleifera* Lam (Moringaceae)**	cultivated	available species	glucose, fructose [103]	antiobesity and hypolipidemic activity [104]
***43. Ozoroa insignis* Del. (Anacardiaceae)**	wild	available species	methyl 3α,24*S*-dihydroxytirucalla-8,25-dien-21-oate; methyl 3α-hydroxy-24-oxotirucalla-8,25-dien-21-oate [105]	antimicrobial activity, cytoprotective effect [106]
***44. Panicum laetum* Kunth (Poaceae)**	wild	available species	proteins, carbohydrates [107]	
***45. Parkia biglobosa* (Jacq.) G.Don (Fabaceae)**	wild	threatened species	cardiac glycosides, steroids, tannins and alkaloids [108]	antiplasmodial activities [109] the antisnake venom activities [110]
***46. Phoenix dactylifera* L. (Arecaceae)**	wild	available species	carbohydrate, vitamins, proteins [111]	nephroprotective, antibacterial, antidiabetic activities [111]
***47. Pseudocedrela kotschyi* (Schweinf.) Harms (Meliaceae)**	wild	available species	tannins, saponins [112]	nephroprotective activities [113]
***48. Raphionacme daronii* Berhaut (Asclepiadaceae)**	wild	available species	sugars and starch [114].	
***49. Saba senegalensis* (A.DC.) Pichon var. *senegalensis (*Apocynaceae)**	wild	threatened species	malic acid, protein, vitamin c [115], tannins, flavonoids, saponins, coumarins, anthocyanosides, triterpenes and sterols [116]	anti-inflammatory, analgesic effect [116]
***50. Sarcocephalus latifolius (*Sm.) E.A.Bruce (Rubiaceae)**	wild	available species	21-*O*-ethylstrictosamide aglycone, strictosamide, angustine, nauclefine, angustidine, angustoline, 19-*O*-ethylangustoline, naucleidinale, 19-epi-naucleidinale [117]	anti-microbial activities [118]
***51. Sclerocarya birrea* (A. Rich.) Hochst. (Anacardiaceae)**	wild	available species	cellulose, proteins, [119] anthocyanins, flavonoids, tannins, saponins [120]	hypoglycemic activity [121]
***52. Sterculia setigera* Delile (Sterculiaceae)**	wild	threatened species	saponins, steroidal, sterols and flavonoids [122]	antiplasmodial, anti-inflammatory activity [123]
***53. Strychnos spinosa* Lam. Lam (Loganiaceae)**	wild	available species	saringosterol and 24-hydroperoxy-24-vinylcholesterol [124]	antitrypanosomal activity [125]
***54. Tamarindus indica* L. (Fabaceae)**	wild	threatened species	9β, 19-Cyclo-4 β4, 4, 14, x-trimethyl-5α-cholestan-3β-ol, 24*R*-Ethyl cholest-5-ene [126]	antiobesity effect [127]
***55. Terminalia avicennioides* Guill. & Perr. (Combretaceae)**	wild	available species	steroids, glycosides, flavonoids, tannins, ellagic acids arjunolic acid, α-amyrin, 2,3,23-trihydroxylolean-12-ene [128]	antimycobacterial activty [128]
***56. Terminalia* macroptera Guill. & Perr. (Combretaceae)**	wild	threatened species	3,3’di-*O*-methylellagic acid, 3,4,3’,4’-tetra-*O*-methylellagic acid, terflavine A [129]	anti-helicobacter pylori activity [130]
***57. Vernonia kotschyana* Sch.Bip. ex Walp. (Astéraceae)**	wild	threatened species	arabinogalactane pectin [131], vernoniosides D1, D2, D3, F1 and F2 and a new androst-8-ene glucoside [132]	antiulcer activity [133]
***58. Vitellaria paradoxa* C.F.Gaertn. (Sapotaceae)**	wild/cultivated	threatened species	anthocyanins, flavonoids, catechol tannins, saponins [134]	emmenagogue [134]
***59. Vitex doniana* Sweet (Verbenaceae)**	wild	threatened species	flavonoids, anthracene derivatives, essential oil, pigments, tannins, terpenes glycosides, triterpenes [135]	antimicrobial activities [136]
***60. Ximenia americana* L. (Olacaceae)**	wild	threatened species	triterpen (mediagenic acid; oleanen glucoside) and steroidal compounds (6–7 hydrositosteron;sitosteroside) [137]	antimicrobial, antitrypanosomal, molluscicide and analgesic [137]
***61. Xysmalobium heudelotianum* Decne. (Asclepiadaceae)**	wild	threatened species		
***62. Zizyphus mauritiana* Lam. (Rhamnaceae)**	wild	threatened species	tannins, sterols and triterpenes, flavonoids, leucoanthocyanins [138]	anti hyperglycemic activities, antihypertensive, and diuretic activity [139]
